# Two Cases of Surreptitious Steroid Use Uncovered Utilizing Urine Synthetic Glucocorticoid Testing

**DOI:** 10.1210/jcemcr/luad132

**Published:** 2023-11-08

**Authors:** Bryn J Pape, Philip A Kern

**Affiliations:** Division of Endocrinology, Department of Internal Medicine, University of Kentucky, Lexington, KY 40504, USA; Division of Endocrinology, Department of Internal Medicine, University of Kentucky, Lexington, KY 40504, USA

**Keywords:** cushing syndrome, factitious, surreptitious steroids, synthetic glucocorticoid testing

## Abstract

Although most patients are transparent regarding steroid use, rare patients use steroids surreptitiously, which can occasionally result in factitious Cushing syndrome or extensive diagnostic testing. We present 2 cases, 1 with factitious Cushing syndrome and the second with surreptitious steroid use resulting in abnormal laboratory results and a complicated clinical picture. Synthetic glucocorticoid urine testing was positive for triamcinolone acetonide and fluticasone propionate in case 1 and triamcinolone acetonide only in case 2, which clarified the diagnosis and minimized additional and potentially invasive testing.

## Introduction

Corticosteroids are commonly prescribed and used for many conditions. Cushing syndrome from pituitary, adrenal, or ectopic tumors are classic, but rare. Whereas iatrogenic Cushing syndrome is a common occurrence resulting from the use of supraphysiologic doses of steroids. Factitious Cushing syndrome or abnormal adrenal function tests that result from the surreptitious use of steroids are even rarer. Here we present 2 such cases, 1 of factitious Cushing syndrome and the other with abnormal adrenal laboratory results from surreptitious steroid use, where we used urine synthetic glucocorticoid screening to facilitate diagnosis.

## Case Presentation

Case 1 is a woman in her late 20s who presented to the outpatient clinic for concerns of unintentional weight gain, increased facial hair, heavy and irregular menstrual cycles, abdominal stretch marks, and diffuse joint pain. The patient had a history of hypothyroidism, depression, and substance abuse. Her reported medications were levothyroxine, bupropion, and methadone. On examination, the patient was hypertensive (blood pressure 152/82 mm Hg) and obese (weight, 146 kg; body mass index, 47.4). Her documented weight 1 year earlier was 60 kg. The patient had facial plethora, hirsutism, central obesity with 1-cm-wide purple striae diffusely on abdomen, thighs, and arms, as well as marked pitting edema to her knees. She had an average to above average health literacy and denied any steroid use in the past year.

Case 2 is a woman in her mid-30s who was admitted for recurrent abdominal pain, nausea, vomiting, and failure to thrive. She had a medical history of chronic pancreatitis, anorexia, alcohol use disorder, and multiple hospitalizations over the past several years for gastrointestinal symptoms and failure to thrive, resulting in insertion of a port for chronic use of total parenteral nutrition. The patient was thin, not overtly cushingoid, and in no acute distress. She had an above average health literacy and knowledge of medical terminology. Throughout her admission, her blood pressure was consistently at or above 120/80 mm Hg. Her recorded weight was 40.8 kg (body mass index, 17.6). The patient denied any steroid use for the past year, although she admitted in years past occasional use of nasal spray for allergies. Full review of medications showed no steroid administration during her hospital stay.

## Diagnostic Assessment

Case 1. Because of the patient's cushingoid appearance, laboratory tests were drawn including a random cortisol, ACTH, and dehydroepiandrosterone sulfate (DHEAS), all of which were suppressed, suggesting exogenous steroid use. On further questioning, the patient acknowledged that she had received an injection, presumably a steroid, in her foot and hip within the 4 months previously. She was asked to refrain from any steroid use. Repeat laboratory tests were obtained 4 months later, again showing suppressed cortisol at <55.2 nmol/L (<2 µg/dL) (reference range [RR], 165.6-690 nmol/L [6-25 µg/dL]) and ACTH <0.66 pmol/L [<3 pg/mL] (RR, 1.58-13.86 pmol/L [7.2-63 pg/mL]) with a suppressed 24-hour urine free cortisol of <0.009 mmol/24 h (< 1.0 mg/24 h) (RR, ≤ 0.41 mmol/24 h [≤45 µg/24 h]). These results remained consistent with exogenous steroid use. A urine sample for synthetic glucocorticoid screen was sent to Mayo Clinic Labs. Results showed that the patient was strongly positive for triamcinolone acetonide, a corticosteroid about 8 times as potent as prednisone used topically, orally, and via injection, and fluticasone, a commonly used variable potency corticosteroid given via oral, inhaled, nasal, or topical routes.

Case 2. This patient had workup for adrenal insufficiency with cosyntropin stimulation testing multiple times in the 3 years before her current hospitalization. Her baseline cortisol and ACTH levels were always exceptionally low or unmeasurable, but her cortisol levels would stimulate normally following cosyntropin injection. During this admission, her baseline cortisol was low at 63.48 nmol/L (2.3 µg/dL) (RR, 165.6-690 nmol/L [6-25 µg/dL]), ACTH was unmeasurable at <0.66 pmol/L (<3 pg/mL) (RR, 1.58-13.86 pmol/L [7.2-63 pg/mL]), and DHEAS was low at 21.79 nmol/L (6.3 µg/dL) (RR, 210.6-1373.2 nmol/L [60.9-397 µg/dL]). Following cosyntropin stimulation, her cortisol increased appropriately to 690 nmol/L (25 mcg/dL) (RR, 165.6-690 nmol/L [6-25 µg/dL]). Although this patient was receiving morphine for her chronic pain, it is doubtful that she received enough to cause secondary adrenal insufficiency, because opioid doses were low, and her urine drug screen did not detect any nonprescribed opioids. The adrenal laboratory results were more suspicious for exogenous steroid use. A random urine sample was sent for synthetic glucocorticoid screen to Mayo Clinic Labs.

## Treatment

While awaiting the results of the urine synthetic glucocorticoids screen, the patient in case 2 was started on hydrocortisone at physiological dosing (15 mg in the morning and 5 mg in the evening daily) because of concern for possible tertiary adrenal insufficiency. However, there was no improvement in symptoms after 5 days, and combined with the normal adrenal stimulation testing, it was felt adrenal insufficiency was unlikely to be the cause of this patient's symptoms and abnormal laboratory results.

The urine synthetic corticosteroid screening test showed that the patient was positive for triamcinolone acetonide.

## Outcome and Follow-up

Case 1. The patient's symptoms and cushingoid appearance were likely from multiple exogenous steroids, although the full extent of which steroids she was using remained unclear. A review of pharmacy records showed scattered prescriptions of prednisone for short courses over the past year but none for triamcinolone acetonide. When presented with this information, the patient admitted to daily inhaled steroids and occasional use of prednisone for asthma but continued to deny any other steroid use.

It was recommended that the patient avoid exogenous steroids with plans to follow up in the clinic for further testing. Unfortunately, the patient did not follow up as scheduled and further testing has not been completed.

Case 2. The urine sample was positive for triamcinolone acetonide, a potent corticosteroid used topically, orally, and via injection to treat various skin, lung, and joint conditions though no records of this medication being administered were found. The patient continued to deny any home steroid use and was discharged with recommendations to avoid exogenous steroids with plans for outpatient follow up. Unfortunately, the patient was subsequently readmitted and died from complications of her continued use of nonmedically prescribed substances related to a generalized factitious disorder.

## Discussion

Cushing syndrome results from chronically elevated levels of glucocorticoids. Endogenous Cushing syndrome has an estimated incidence of 0.7 to 2.4 per million population per year, with the most common cause arising from pituitary overproduction of ACTH [1]. Distinctive symptoms and signs include a dorsocervical fat pad, moon facies, purple striae, plethora, proximal muscle weakness, and easy bruising, along with features common in the general population such as obesity, depression, diabetes, hypertension, and menstrual irregularities. The use of exogenous steroids is the most common cause of cushingoid clinical features [2].

It is usually straightforward to determine if Cushing syndrome is endogenous or exogenous because patients, and their medical records, are usually transparent regarding steroid use. Endocrine Society guidelines recommend “a thorough drug history” of steroid medications before performing laboratory testing [2]. The workup for endogenous Cushing syndrome includes screening tests, followed by additional studies to determine if cortisol is ACTH-dependent, and ultimately imaging of the pituitary, adrenals, or sampling from the inferior petrosal sinus or adrenal veins [1, 2]. At initial presentation, a plasma DHEAS level is useful because this adrenal hormone is ACTH responsive and would be low with chronic suppression of ACTH from an adrenal or exogenous source of steroid.

Factitious disorder (FD) is a psychiatric disorder in which the patient fabricates illness to undergo medical care without any obvious material gain. The patient's motivation can be obscure and arise from a desire for affection or to provide a sense of control. The incidence of FD is unknown because it is often undiagnosed [3]. A previous review found that most patients with FD were female, with a mean age of 34 years, often worked in health care, and often had another psychiatric disorder such as depression, substance abuse, anxiety, and eating disorders. FD cases within endocrinology were reported 59 times, with the most common presentation of hypoglycemia from exogenous antidiabetic medications [3].

Factitious Cushing syndrome resulting from a surreptitious use of exogenous steroids is rare, with only 9 reported cases in the past 20 years [3]. One case, reported in 2006, involved a 33-year-old female who died from invasive pulmonary aspergillosis before it was determined she was taking daily prednisone for years, resulting in atrophied adrenal glands and cushingoid features [4]. No other deaths from factitious Cushing syndrome were found in our literature review.

Factitious Cushing syndrome can present with typical symptoms and low levels of cortisol and ACTH. However, standard clinical assays for cortisol will not detect most synthetic steroids, and therefore factitious use can be hard to prove, resulting in additional workup and medical care expenses. For example, Pineyro et al reported a 26-year-old woman with cushingoid features who underwent several invasive procedures, including unilateral adrenalectomy, before synthetic glucocorticoid testing eventually disclosed elevated levels of serum prednisone and prednisolone [5].

The Mayo Clinic has established serum and urine laboratory screening tests for synthetic glucocorticoids, both using liquid chromatography tandem mass spectrometry Stable Isotope Dilution Analysis. The urine screening test can detect betamethasone, budesonide, dexamethasone, fludrocortisone, fluticasone propionate, megestrol acetate, methylprednisolone, prednisolone, prednisone, and triamcinolone acetonide. The serum screening test can detect all the same synthetic glucocorticoids as the urine screening test except for fluticasone propionate. Both tests use negative cutoff values of 0.1 mcg/dL for all tested glucocorticoids, except for budesonide, which has a cutoff value of 0.2 mcg/dL [6].

Our cases represent patients who were surreptitiously taking steroids that resulted in either cushingoid features or abnormal adrenal testing. Utilization of synthetic glucocorticoid screening helped avoid further invasive workup, but the outcomes were unfortunately poor. In case 1, the patient had classical Cushing syndrome features and positive urine glucocorticoid screening but was lost to follow up. In case 2, the patient presented with a complex medical disorder but did not have typical cushingoid signs or symptoms. Her gastrointestinal symptoms and failure to thrive were concerning for adrenal insufficiency but because she did not respond to steroid treatment, had normal adrenal stimulation, and a positive urine synthetic glucocorticoid screen, she was more likely to have surreptitious steroid use. The patient ultimately passed away from complications linked to her factitious disorder, although it is unknown what role steroid use played in her death.

There are several cases reported where synthetic glucocorticoid screening was used either during or after the work up for Cushing syndrome. Anderson et al presented 2 patients with cushingoid symptoms, both denying any exogenous steroid use, where HPLC urine testing showed prednisone or prednisolone [7]. Determination of synthetic glucocorticoids by HPLC was also used to diagnosis 4 patients with factitious Cushing syndrome from 860 patients referred for hypercortisolism to the National Institute of Health Clinical Center over a 15-year period [8].

Although the true occurrence of factitious Cushing syndrome or surreptitious steroid use is unknown, the use of screening for synthetic glucocorticoids in patients presenting with Cushing syndrome may be useful in suspicious cases, such as where adrenal laboratory tests are inconclusive before pursuing more invasive testing or procedures. As seen in our cases, and those in the literature, the use of exogenous steroids may not be easily elicited during history taking. The use of urine synthetic glucocorticoid screening could relieve a burden on the health care system and save patients’ lives.

## Learning Points

- Surreptitious glucocorticoid use can cause cushingoid symptoms and/or abnormal adrenal laboratory results.
- Screening for synthetic glucocorticoids in urine or serum can facilitate diagnosis of factitious Cushing syndrome.
- Diagnosis of factitious Cushing syndrome early can eliminate unnecessary invasive testing and procedures.

## Contributors

All authors made individual contributions to authorship. B.P. and P.K. were involved in the diagnosis and management of patients and manuscript submission. All authors reviewed and approved the final draft.

## Data Availability

Data sharing is not applicable to this article as no datasets were generated or analyzed during the current study.

## Abbreviations

DHEAS: dehydroepiandrosterone sulfate
FD: factitious disorder
RR: reference range
